# The role of diabetes co-morbidity for tuberculosis treatment outcomes: a prospective cohort study from Mwanza, Tanzania

**DOI:** 10.1186/1471-2334-12-165

**Published:** 2012-07-27

**Authors:** Daniel Faurholt-Jepsen, Nyagosya Range, George Praygod, Jeremiah Kidola, Maria Faurholt-Jepsen, Martine Grosos Aabye, John Changalucha, Dirk Lund Christensen, Torben Martinussen, Henrik Krarup, Daniel Rinse Witte, Åse Bengård Andersen, Henrik Friis

**Affiliations:** 1 Department of Human Nutrition, University of Copenhagen, Frederiksberg, Denmark; 2 Muhimbili Medical Centre, National Institute of Medical Research, Dar Es Salaam, Tanzania; 3 Mwanza Medical Centre, National Institute of Medical Research, Mwanza, Tanzania; 4 Clinical Research Centre, University of Copenhagen, Hvidovre Hospital, Hvidovre, Denmark; 5 Department of International Health, University of Copenhagen, Copenhagen, Denmark; 6 Steno Diabetes Center, Gentofte, Denmark; 7 Department of Basic Sciences and Environment, University of Copenhagen, Frederiksberg, Denmark; 8 Department of Clinical Biochemistry, Aalborg University Hospital, Aalborg, Denmark; 9 Department of Infectious Diseases, Odense University Hospital, Odense, Denmark

**Keywords:** Tuberculosis, Diabetes, Treatment outcome, Anthropometry, Haemoglobin, Grip strength

## Abstract

**Background:**

Due to the association between diabetes and pulmonary tuberculosis (TB), diabetes may threaten the control of TB. In a prospective cohort study nested in a nutrition trial, we investigated the role of diabetes on changes in anthropometry, grip strength, and clinical parameters over a five months follow-up period.

**Methods:**

Among pulmonary TB patients with known diabetes status, we assessed anthropometry and clinical parameters (e.g. haemoglobin) at baseline and after two and five months of TB treatment. A linear mixed-effects model (repeated measurements) was used to investigate the role of diabetes during recovery.

**Results:**

Of 1205 TB patients, the mean (standard deviation) age was 36.6 (13.0) years, 40.9% were females, 48.9% were HIV co-infected, and 16.3% had diabetes. TB patients with diabetes co-morbidity experienced a lower weight gain at two (1.3 kg, CI95% 0.5; 2.0, p = 0.001) and five months (1.0 kg, CI95% 0.3; 1.7, p = 0.007). Similarly, the increase in the level of haemoglobin was lower among TB patients with diabetes co-morbidity after two (Δ 0.6 g/dL, CI95% 0.3; 0.9 p < 0.001) and five months (Δ 0.5 g/dL, CI95% 0.2; 0.9 p = 0.004) of TB treatment, respectively.

**Conclusion:**

TB patients initiating TB treatment with diabetes co-morbidity experience delayed recovery of body mass and haemoglobin, which are important for the functional recovery from disease.

## Background

Chronic and infectious diseases often co-exist due to mutual risk factors as well as direct interactions between the diseases. One of the major challenges is the double burden of diabetes and pulmonary tuberculosis (TB) [1]. With the on-going diabetes epidemic in low-income countries already burdened by TB [2], diabetes may threaten the control of TB.

Previous studies have found that diabetes may impair sputum conversion and cure [3-5] and increase the risk of relapse [6]. However, the studies focus on the treatment outcome related to clearance of the TB bacteria and do not take into account the impact of diabetes on the outcome of other factors such as body composition, functional recovery, as well as other clinical parameters affected by disease. We have previously demonstrated a 10 kg weight deficit among newly diagnosed TB patients compared to non-TB individuals [7], thus the regain of lean and fat mass is an important part of TB recovery, since rapid build-up of fat mass may lead to metabolic syndrome [8]. Also, TB patients often experience inflammation-induced anaemia as a consequence of the TB disease [9], thus a rise in haemoglobin during TB treatment can be considered a marker of recovery. We recently reported from Tanzania that while diabetes was associated with a higher risk of TB [10], diabetes had little consequences for the clinical manifestations of newly diagnosed TB [11]. In this paper we report the changes in anthropometry, grip strength, and clinical parameters over a five months follow-up period, to assess the role of diabetes on recovery during TB treatment.

## Methods

### Study population and design

From 2006 to 2008 patients newly diagnosed with active pulmonary TB were consecutively enrolled in this prospective cohort study in the framework of two large randomized, double-blind, controlled nutrition intervention studies, with all patients diagnosed and treated for TB according to international guidelines [12]. All participants were randomized to either an energy-protein study [13] or a multi-micronutrient study [14] comparing the effect of high-doses against low-doses. To be eligible for the energy-protein study, TB patients had to be diagnosed as sputum positive pulmonary TB (PTB+) with HIV co-infection. All other enrolled TB patients were eligible for the multi-micronutrient study.

The study was conducted in an urban setting in Mwanza City, northern Tanzania. Tanzania ranks among the world’s 22 high TB-burden countries [15], with a low prevalence of multi-drug resistant TB [16], and half of the TB patients co-infected with HIV [17]. The estimated national diabetes prevalence in 2011 in Tanzania was 2.3% [18]. All participants underwent baseline examination of anthropometry, diabetes, and HIV, and venous blood samples were drawn for additional laboratory analyses. To be included in the study testing for HIV was mandatory. Persons below fifteen years of age, pregnant or lactating women, terminally ill, and non-residents of Mwanza City were all excluded.

### Measurements

Sputum results were done as part of the routine diagnostic procedure using “spot-morning-spot” samples (method described in [12]) using Ziehl-Nielsen staining technique in combination with culture of *Mycobacterium tuberculosis* on Lowenstein Jensen solid media. All participants had verified pulmonary TB (PTB); in this study a positive culture test result was defined as PTB + with the diagnosis relying primarily on culture status; initial microscopy results were only used if the culture result was missing. In case of a negative culture result, the diagnosis was defined as sputum negative pulmonary TB (PTB-), in which case the TB diagnosis was based on clinical suspicion, history of disease, lack of clinical improvement after treatment with a broad antibiotic spectrum as well as a positive x-ray result as suggested by WHO [19].

Weight (Seca, Hamburg, Germany) and height were measured with the participant barefoot and with minimal clothing (nearest 0.1 kg and 0.1 cm), from which body mass index (BMI) was calculated as weight/height^2^ (kg/m^2^). Waist circumference was measured between the lower costae and the iliac crest. The midpoint between the acromion process of the shoulder and olecranon process of the ulna bone was determined and marked on the left arm, on which mark the triceps skinfold thickness (TST) (Harpenden caliper, Baty International, West Sussex, UK) was measured (with arm hanging loosely). Mid-upper arm circumference (MUAC) was measured on the same arm and same mark using a standard tape, but with the arm flexed in a 90^°^ angle. Measuring TST and MUAC allowed for estimation of arm fat area and arm muscle area (methods for calculation described in [20]). Finally, grip strength (0.1 kg) was assessed using a digital hand dynamometer (Takei Scientific Instruments, Niigata City, Japan). All anthropometric measurements were performed in duplicate.

Fasting blood glucose (FBG) was determined on capillary whole blood using point-of-care diagnostic instruments (HemoCue Glucose System, Ängelholm, Sweden). The test was performed between 8.00-10.00 AM after an overnight fasting period (> 8 hours), and only water was allowed prior to the test. As the FBG in the TB participants might be affected by the infection (non-diabetes stress hyperglycaemia) [21,22], the range of the FBG for offering a standard two hour (2 h) oral glucose tolerance test (OGTT) was expanded from the commonly used 5.6-6.0 mmol/L; those with a FBG between 5.1-11.0 mmol/L completed the 2 h OGTT (intake of 75 g of anhydrous glucose dissolved in water), whereas those with FBG < 5.1 or >11.0 mmol/L did not. Final diabetes diagnosis was based on either a FBG > 6.0 mmol/L or a 2 h blood glucose >11.0 mmol/L [23]. Since the diagnosis of diabetes was for epidemiological purposes only, we did not repeat the test in those with values suggestive of diabetes. Participants diagnosed with diabetes prior to their TB diagnosis were only classified as such, if the diabetes diagnosis was reproduced within the present study. The diabetes testing was performed as soon as possible after initiation of TB treatment to eliminate the role of adverse drug effects.

Venous blood was drawn in EDTA tubes at local health facilities and transported to the research laboratory, whereupon serum was collected and kept at −80 °C until analysed. HIV diagnosis was based on two rapid tests, Determine HIV 1/2 (Inverness Medical Innovations Inc., Delaware, USA) and Capillus HIV-1/HIV-2 (Trinity Biotech Plc., Wicklow, Ireland). If the HIV test results were discordant, ELISA was used. Cluster of differentiation 4 (CD4) counts were determined by flow cytometry after CD4 immuno-flourochrome staining of the leucocytes (Partec FACS, Partec GmbH., Germany), and haemoglobin levels (g/dL) and white blood cell (10^9^/L) counts, including differentials, were carried out at the research laboratory at the National Institute for Medical Research in Mwanza. Serum concentrations (g/L) of the acute phase reactant alpha-1-acid glycoprotein were determined at the Department of Clinical Biochemistry, Aalborg University Hospital, Denmark.

Information using standardized questionnaires on demographic information, smoking habits, and alcohol intake was collected. Smokers were grouped as either previous or current smokers, and alcohol intake was classified as either no intake or any intake.

### Follow-up visits at two and five months

All anthropometric measurements, grip strength and the biological measurements (haemoglobin, white blood cells, CD4) were repeated at the two and five months visit. The diabetes and HIV testing were not repeated, thus the baseline diagnosis was used throughout the study period.

### Statistical analysis

Data were double entered, and all statistical analyses were performed using Stata 12.0 (StataCorp LP, College Station, USA). The distribution of continuous variables was assessed for normality. The t-test was used to test for differences in means and the *Χ*^2^-test was used to test for differences in proportions across diabetes status. A linear mixed-effects model [24] was used to assess the changes across diabetes status for anthropometry, grip strength and biological measurements at baseline as well as after two and five months of TB treatment (repeated measurements). The mixed-effects models were adjusted for baseline age, sex, HIV status, alpha-1 glycoprotein, smoking habits, alcohol intake, and nutritional intervention.

### Ethical considerations

Ethical permission was obtained from the Medical Research Coordinating committee of the National Institute for Medical Research (NIMR) in Tanzania, and consultative approval was given by The Danish National Committee on Biomedical Research Ethics. Written and oral information was presented to all eligible participants by the health staff before written informed consent was obtained. Written consent was obtained from parents/legal guardians of any participants under 18 years of age. Counselling prior to HIV-testing was compulsory, and post-test counselling was offered to all who tested HIV-positive. Participants with diagnosed HIV and/or diabetes were referred for follow-up at the respective clinics for care and management.

## Results

Diabetes data were available for 1205 (96.4%) of the 1250 patients enrolled and included in the analyses. Of the 1205 TB patients 197 (16.3%) were categorized as having diabetes, which has previously been reported for PTB + patients only (n = 803) [10]. The mean (standard deviation) age was 36.6 (13.0) years, 40.9% were females, and 48.9% were HIV co-infected. There were no differences in background characteristics between TB patients with and without diabetes (Table 1).

**Table 1:** Background characteristics of 1205 pulmonary tuberculosis patients with (n = 197) or without (n = 1008) diabetes

	**Patients without diabetes**	**Patients with diabetes**	**p**
	**(n = 1008)**	**(n = 197)**	
Age, years (mean [SD])	36.3 [12.8]	38.0 [13.6]	0.083
Female sex	404 (40.1)	89 (45.2)	0.183
HIV infection	507 (50.3)	97 (49.2)	0.786
TB status			
PTB-	339 (33.6)	63 (32.0)	0.65
PTB+	669 (66.4)	134 (68.0)	
Ethnic group			
Msukuma tribe	459 (45.6)	94 (47.7)	0.582
Other tribes	548 (54.4)	103 (52.3)	
Marital status			
Single	249 (24.9)	42 (21.5)	0.605
Married/cohabiting	528 (52.8)	108 (55.4)	
Separated/divorced/widow	223 (22.3)	45 (23.1)	
Occupation			
Farmer/Fisherman	394 (39.2)	75 (38.3)	0.974
Businessman/Employed	356 (35.4)	74 (37.8)	
Housewife	120 (11.9)	23 (11.7)	
Unemployed	51 (5.1)	9 (4.6)	
Religion			
Christian	741 (73.6)	155 (78.7)	0.191
Muslim	223 (22.1)	38 (19.3)	
Smoking			
Never	675 (82.9)	139 (17.1)	0.60
Past smoker	110 (85.9)	18 (14.1)	
Current smoker	216 (84.7)	39 (15.3)	
Take alcohol			
No	533 (82.9)	110 (17.1)	0.45
Yes	475 (84.5)	87 (15.5)	

Data are n(%) unless otherwise specified.

**Figure 1:**
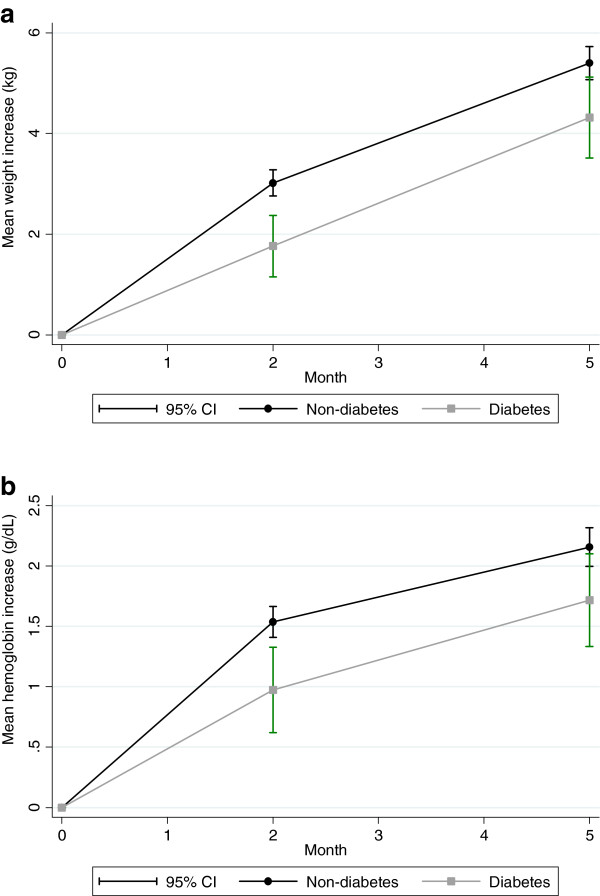
**a Changes in weight level during treatment among pulmonary tuberculosis patients with (n = 197) or without (n = 1008) diabetes.** Figure 1**b**. Changes in haemoglobin level during treatment among pulmonary tuberculosis patients with (n = 197) or without (n = 1008) diabetes.

The changes in anthropometric measurements during TB treatment are shown in table 2. Within the initial two months of TB treatment, TB patients with diabetes co-morbidity experienced a 1.3 kg (CI 95% 0.5; 2.0, p = 0.001) lower weight gain compared to the non-diabetes group. The delayed weight gain sustained at five months of TB treatment, with a 1.0 kg (CI 95% 0.3; 1.7, p = 0.007) lower weight gain among TB patients with diabetes co-morbidity (Table 2, Figure 1a). There was no baseline difference in the mean MUAC (22.9 vs. 23.3 cm, p = 0.185) and TST (7.2 vs. 7.3, p = 0.581) between those with and without diabetes. However, the increase in MUAC was higher in the non-diabetes group after two (Δ 0.3 g/dL, CI 95% 0.04; 0.6 p = 0.027) and five months (Δ 0.3 g/dL, CI 95% 0.02; 0.6 p = 0.036), whereas no difference was found for TST (two months: Δ 0.3 g/dL, CI 95% -0.2; 0.8 p = 0.288 and five months: Δ 0.3 g/dL, CI 95% -0.2; 0.8 p = 0.283). Although significant increases were seen for arm muscle area and arm fat area for both groups, there were no differences across diabetes status. Similarly, there were significant increases for waist circumference and grip strength, but the increase seemed to be unaffected by diabetes status.

**Table 2:** Changes in anthropometric measurements and grip strength during TB treatment among TB patients with (n = 197) or without (n = 1008) diabetes

	**Patients without diabetes**	**Patients with diabetes**	
	**(n = 2008)**	**(n = 197)**	
	**Mean (95% CI)**	**Mean (95% CI)**	**P**
Weight, kg			
Baseline	51.8 (51.3; 52.3)	53.3 (52.1; 54.5)	
2 months	54.8 (54.3; 55.3)	55.1 (53.9; 56.3)	
Increase	3.0 (2.7; 3.3)	1.7 (1.1; 2.4)	
Difference	1.3 (0.5; 2.0)		0.001
5 months	57.1 (56.6; 57.7)	57.7 (56.4; 58.9)	
Increase	5.3 (5.0; 5.6)	4.3 (3.7; 5.0)	
Difference	1.0 (0.3; 1.7)		0.007
BMI, kg/m^2^			
Baseline	18.7 (18.6; 18.9)	19.3 (18.9; 19.7)	
2 months	19.8 (19.6; 20.0)	19.9 (19.5; 20.3)	
Increase	1.1 (1.0; 1.2)	0.6 (0.4; 0.9)	
Difference	0.4 (0.2; 0.7)		0.001
5 months	20.7 (20.5; 20.9)	20.9 (20.4; 21.3)	
Increase	1.9 (1.8; 2.0)	1.6 (1.3; 1.8)	
Difference	0.3 (0.1; 0.6)		0.012
Waist circumference, cm			
Baseline	72.1 (71.7; 72.5)	73.7 (72.8; 74.7)	
2 months	74.5 (74.0; 74.9)	75.8 (74.8; 76.9)	
Increase	2.4 (2.1; 2.7)	2.1 (1.4; 2.8)	
Difference	0.3 (−0.5; 1.0)		0.512
5 months	76.3 (75.9; 76.8)	77.8 (76.8; 78.8)	
Increase	4.2 (3.9; 4.5)	4.1 (3.3; 4.8)	
Difference	0.2 (−0.6; 1.0)		0.697
AMA, mm^2^			
Baseline	34.6 (34.0; 35.1)	35.6 (34.3; 37.0)	
2 months	37.2 (36.6; 37.8)	37.4 (36.0; 38.7)	
Increase	2.7 (2.2; 3.1)	1.7 (0.7; 2.7)	
Difference	0.9 (−0.2; 2.0)		0.100
5 months	40.7 (40.1; 41.4)	41.0 (39.6; 42.4)	
Increase	6.2 (5.7; 6.6)	5.4 (4.4; 6.4)	
Difference	0.8 (−0.3; 1.9)		0.164
AFA, mm^2^			
Baseline	8.1 (7.8; 8.4)	8.3 (7.6; 9.0)	
2 months	9.2 (8.9; 9.5)	9.0 (8.3; 9.8)	
Increase	1.1 (0.9; 1.4)	0.8 (0.1; 1.4)	
Difference	0.4 (−0.3; 1.0)		0.297
5 months	10.4 (10.1; 10.8)	10.4 (9.6; 11.2)	
Increase	2.3 (2.1; 2.6)	2.1 (1.5; 2.8)	
Difference	0.2 (−0.5; 0.9)		0.524
Grip strength, kg			
Baseline	25.8 (25.3; 26.3)	25.2 (24.2; 26.2)	
2 months	27.6 (27.2; 28.1)	26.8 (25.7; 27.9)	
Increase	1.8 (1.5; 2,2)	1.6 (0.7; 2.4)	
Difference	0.2 (−0.7; 1.2)		0.610
5 months	30.5 (30.0; 30.9)	29.6 (28.5; 30.7)	
Increase	4.7 (4.3; 5.0)	4.4 (3.5; 5.2)	
Difference	0.3 (−0.7; 1.2)		0.550

Data are mean (95% confidence interval) based on multilevel mixed-effects linear regression, adjusted for age, sex, HIV status, alpha-1 glycoprotein, smoking habits, alcohol intake, and nutritional intervention.

TB: pulmonary tuberculosis, BMI: body mass index, AMA: arm muscle area, AFA: arm fat area.

The mean haemoglobin level was 10.7 g/dL at baseline, and was similar for non-diabetes and diabetes participants (Δ 0.03 g/dL, p = 0.851). However, the increase in haemoglobin was 0.6 g/dL (CI 95% 0.3; 0.9 p < 0.001) and 0.5 g/dL (CI 95% 0.2; 0.9 p = 0.004) lower after two and five months, respectively, among TB patients with diabetes co-morbidity (Table 3, Figure 1b). Diabetes did not affect the changes in the level of white blood cells and CD4 counts, and this was not modified by HIV status (data not shown).

**Table 3:** Changes in haemoglobin level, white blood cell and CD4 count during treatment among pulmonary tuberculosis patients with (n = 197) or without (n = 1008) diabetes

	**Patients without diabetes**	**Patients with diabetes**	
	**(n = 2008)**	**(n = 197)**	
	**Mean (95% CI)**	**Mean (95% CI)**	**P**
Haemoglobin, g pr. dL			
Baseline	10.8 (10.6; 10.9)	10.7 (10.4; 11.0)	
2 months	12.3 (12.2; 12.5)	11.7 (11.4; 12.0)	
Increase	1.6 (1.4; 1.7)	1.0 (0.7; 1.3)	
Difference	0.6 (0.3; 0.9)		< 0.001
5 months	13.0 (12.8; 13.1)	12.4 (12.1; 12.7)	
Increase	2.2 (2.1; 2.3)	1.7 (1.4; 2.0)	
Difference	0.5 (0.2; 0.9)		0.004
White blood cell count (total), 10^9^ cells pr. L			
Baseline	6.4 (6.3; 6.6)	6.8 (6.5; 7.1)	
2 months	4.7 (4.6; 4.8)	5.0 (4.7; 5.3)	
Increase	−1.7 (−1.9; -1.6)	−1.7 (−2.1; -1.4)	
Difference	0.02 (−0.3; 0.4)		0.918
5 months	4.3 (4.1; 4.4)	4.6 (4.3; 4.9)	
Increase	−2.2 (−2.3; -2.0)	−2.2 (−2.5; -1.8)	
Difference	−0.03 (−0.4; 0.3)		0.889
Neutrophil granulocytes, 10^9^ cells pr. L			
Baseline	4.1 (4.0; 4.2)	4.6 (4.4; 4.8)	
2 months	2.3 (2.2; 2.4)	2.6 (2.4; 2.8)	
Increase	−1.8 (−2.0; -1.7)	−2.0 (−2.2; -1.7)	
Difference	0.1 (−0.2; 0.4)		0.493
5 months	1.8 (1.7; 2.0)	2.2 (2.0; 2.5)	
Increase	−2.3 (−2.4; -2.1)	−2.3 (−2.6; -2.1)	
Difference	0.1 (−0.2; 0.4)		0.658
CD4 count, cells pr. μL			
Baseline	425.9 (408.9; 442.8)	409.2 (370.6; 447.8)	
2 months	477.5 (459.9; 495.2)	435.1 (393.7; 476.5)	
Increase	51.7 (29.7; 73.6)	25.9 (−25.0; 76.8)	
Difference	25.7 (−29.7; 81.2)		0.362
5 months	436.2 (417.7; 454.6)	396.6 (353.7; 439.6)	
Increase	10.3 (−12.3; 32.9)	−12.5 (−64.6; 39.6)	
Difference	22.8 (−33.9; 79.6)		0.431

Data are mean (95% confidence interval) based on a linear mixed-effects model, adjusted for age, sex, HIV status, alpha-1 glycoprotein, smoking habits, alcohol intake, and nutritional intervention.

## Discussion

Diabetes co-morbidity was associated with delayed recovery of weight and haemoglobin level within the first two months of TB treatment, and the accumulated difference persisted after five months. However, the functional recovery based on grip strength assessment was not affected by diabetes.

Being enrolled in a nutritional intervention study ensured that all participants had access to some nutritional support during the first two months of TB treatment. We recently reported that smear-positive TB was associated with a 10 kg weight loss, which was both reflected in large deficits in arm fat area and especially arm muscle area [7], suggesting that nutritional support to facilitate optimal recovery of lean body mass is needed during the treatment. As reported here, there was a considerable delayed weight gain in those with diabetes. On average the participants gained three and five kg over the two and five months treatment period, respectively, but participants with diabetes had a one kg weight deficit compared to the non-diabetes patients at both time points. Interestingly, this weight difference was built up during the first two months, and sustained at the same level after five months. Also MUAC increased at a slower pace in the diabetes group, whereas TST did not. That MUAC and TST did not behave similarly was reflected in the derived parameter arm muscle area, which also seemed to be increasing slower in the diabetes group. This could imply that people with diabetes have a slower muscle build-up, but this was not backed up by the functional measurement grip strength.

The low levels of haemoglobin at baseline are primarily inflammation-induced anaemia from the on-going HIV and TB infections [9,25]. Regardless of the underlying cause, the haemoglobin level is expected to rise during recovery, which we also found with reasonable increases at two and five months. The slower increase in haemoglobin in those with diabetes could be the consequence of sooner improvement in the non-diabetes group. In parallel with the replenishment of iron to haemoglobin, there may also be a direct association between haemoglobin and lean body mass; either caused by mutual factors improving both lean body mass and haemoglobin levels, or local hypoxia due to low haemoglobin may be the delimiting factor in the build-up of lean body mass in the diabetes group.

The differences we found did not depend on TB status, since the diabetes-associated differences were present among both PTB + and PTB- participants. As suggested, the delayed increase in weight gain and haemoglobin levels in the diabetes group could partly be explained by a slower TB recovery. From a study in severely acute malnourished (marasmic) adults it has been shown that the daily increase in body weight during replenishment is app. 6 g per kg body weight [26]. Thus, any delay in TB recovery may interfere with body mass recovery. However, the slower improvement observed in our study could also be a consequence of poorly controlled diabetes, which is known to be associated with protein degradation and leucine oxidation [27,28]. Finally, the association between diabetes and slow recovery could be explained by reverse causality; i.e. those with slower recovery may have more severe TB disease, and this could give rise to non-diabetes stress-hyperglycaemia [21,22], which may have been misclassified as diabetes. However, this is not likely to be a major factor in this study, since we have observed very little, and probably not clinically important, baseline differences in the acute phase response between diabetes and non-diabetes participants [11], indicating similar baseline severity of TB in the two groups, and, furthermore, since the differences observed were not confounded by the acute phase response. We have previously reported fluctuations in the CD4 level during TB treatment with increases over the initial two months of treatment and with a subsequent decrease [29]. However, the data from this study show that the fluctuations were not affected by diabetes co-morbidity.

All patients diagnosed with diabetes were referred to the local diabetes clinics to be retested and treated accordingly. Data on anti-diabetic treatment has not been available for the present study, however, patients with a reconfirmation of diabetes have most likely started out with advice on lifestyle changes, and therefore medical intervention is unlikely to be confounding the data.

## Conclusion

TB patients initiating TB treatment with diabetes co-morbidity may experience slower recovery from their TB disease. While previous studies have primarily focused on treatment outcomes related to the TB culture (or smear) intensity, conversion, cure and mortality, this study looked at general outcomes, such as anthropometry and haemoglobin, which are important for the functional recovery. A quick functional recovery leads to a sooner return to work, and thus, the delay may not only have beneficial health outcomes, but also economically consequences for low-income families burdened by TB.

## Competing interests

The authors have declared that no competing interests exist.

## Authors’ contributions

HF, NR, JC and ÅBA conceived the study. NR, GP, KJ, DFJ, MFJ and MGA implemented the study. DFJ analysed the data and wrote the first draft of the manuscript. All authors contributed to the interpretation of results and commented on drafts and approved the final version. HF (hfr@life.ku.dk) is guarantor of the paper. All authors read and approved the final manuscript.

## Pre-publication history

The pre-publication history for this paper can be accessed here:

http://www.biomedcentral.com/1471-2334/12/165/prepub
